# Correlation between Serum 25-Hydroxyvitamin D Concentration, Monocyte-to-HDL Ratio and Acute Coronary Syndrome in Men with Chronic Coronary Syndrome—An Observational Study

**DOI:** 10.3390/nu15204487

**Published:** 2023-10-23

**Authors:** Ewelina A. Dziedzic, Jakub S. Gąsior, Agnieszka Tuzimek, Marek Dąbrowski, Wacław Kochman

**Affiliations:** 1 Cardiovascular Clinic, Centre of Postgraduate Medical Education, 01-813 Warsaw, Poland; 2 Department of Pediatric Cardiology and General Pediatrics, Medical University of Warsaw, 02-091 Warsaw, Poland; 3 Department of Cardiology, Bielanski Hospital, 01-809 Warsaw, Poland

**Keywords:** vitamin D, 25-hydroxyvitamin D, HDL, monocyte percentage, MHR

## Abstract

Cardiovascular disease (CVD) continues to be the leading cause of death in European men. Atherosclerosis and its clinical consequence, chronic coronary syndrome (CCS), comprise two main elements: dysfunction of lipoprotein metabolism and an important inflammatory component that contributes to the development of complications, including acute coronary syndrome (ACS). Measures of both components are combined in a composite marker called monocyte-to-HDL ratio (MHR). Vitamin D was previously described to influence inflammation processes, and its deficiency influences CVD risk factors. This research describes the differences in MHR and total serum 25-hydroxyvitamin D (25(OH)D) concentration between male patients with different diagnoses of CCS and the correlation between 25(OH)D and MHR in this group. Significant differences were observed between ACS and CCS patients in 25(OH)D and MHR—the highest HDL and serum 25(OH)D concentrations were observed in patients with CCS, whereas the highest value of MHR was observed in patients with STEMI. A significant correlation was observed between 25(OH)D, HDL, and MHR. Due to the significant but small nominal difference in MHR values between groups of patients diagnosed with ACS and CCS, and the possible influence of age and hyperlipidemia status on the differences in vitamin D levels in these groups, this subject requires further well-designed research. The suggested bidirectional relationship between MHR and 25(OH)D and the role of MHR as a predictor of vitamin D status in the body also needs to be verified.

## 1. Introduction

Atherosclerosis, the cause of chronic coronary syndrome (CCS), is an inflammatory disease of the large and medium arteries with focal plaques typical of the disease [1]. Chronic, subclinical, low-level inflammation plays a pivotal role in the initiation, progression, and destabilization of plaques [2]. It also affects restenosis and modifies the effectiveness of percutaneous balloon angioplasty regardless of stent use [3].

Monocyte-to-HDL ratio (MHR), two components with opposite effects on atherosclerosis, is a biomarker of low-grade systemic inflammation that could be used for the identification of patients with CCS exhibiting high risk of complications.

HDL is a known protective factor for cardiovascular diseases (CVD) since the 1960s [4]. Recent epidemic studies confirmed that low HDL level is an independent risk factor for CVD. Furthermore, it was also shown that high HDL is also correlated with CVD risk [5,6]. HDL plays a key role in the cholesterol efflux capacity, where HDL accepts cholesterol from other cells, e.g., macrophages, by altering the cell membrane and intracellular cholesterol reservoir [7]. The mechanisms behind these phenomena are still under research [4] along with their anti-inflammatory properties [8]. Recent data on MHR in CVD in male patients are limited, and they describe a higher MHR in men with CCS [9,10]. Furthermore, this marker was shown to be an independent predictor of CCS diagnosis [11,12] and progression [9,13,14]. Higher MHR was also observed in patients with acute coronary syndrome (ACS) [15,16,17], stent embolus in patients with ACS treated with percutaneous coronary intervention [18], and microvascular angina [19]. Compared to the concentration of C-reactive protein and the neutrophil-to-lymphocyte ratio, MHR was a better predictor of the presence of significant coronary stenosis in patients diagnosed with ST-elevation myocardial infarction and treated with a percutaneous intervention [20]. Furthermore, in another group of patients, this marker was correlated with the diagnosis of coronary artery disease and predicted the severity of this disease more accurately than HDL concentration or the LDL to HDL ratio [21]. The 25-hydroxyvitamin D (25(OH)D) level influences immune [22] and cardiovascular systems [23]; therefore, it is considered as a new CVD risk factor [24,25]. Immune cells not only have the vitamin D receptor (VDR) [26], but they can also convert 25(OH)D to calcitriol (1,25(OH)D), which allows an autonomic regulation of active vitamin D concentration in an inflamed site [27]. Furthermore, 1,25(OH)D stimulates monocyte proliferation and differentiation as well as suppresses the immune response with influence on macrophages [28]. 25(OH)D deficiency in a healthy population was correlated with the pro-inflammatory phenotype of monocytes (increased adhesion to platelets and endothelium) [29]. In the presence of 1,25(OH)D, monocytes produce an immunoglobulin complement with a key function in the innate response. This suggests an inherent defensive mechanism activated by calcitriol [30]. VDR and 1-alpha-hydroxylase are also present in cells of the cardiovascular system [31,32,33,34]. The 25(OH)D level was described to influence classic risk factors for CVD [35,36,37]. In addition, it is involved in the atherogenesis and development of its complications, including ACS [38,39,40].

Although the European Society of Cardiology reported a slight decrease in the incidence of CVD over the last 30 years, the prevalence disparity between both sexes remains notable. It was shown that men, compared to women, suffer and die from CCS significantly more frequently. In the group of male patients younger than 70 years, CVD is the most common cause of morbidity, with 44% solely due to CCS, followed by stroke [41]. The results of our recent studies indicate a significantly higher value of subclinical inflammation markers based on blood cell elements in patients with myocardial infarction [42] and their negative correlation with total serum 25(OH)D concentration [43]. Thus, the subject of the present study was to analyze MHR values and serum vitamin D levels in a cohort of male patients with coronary artery disease. The aim of the study was to verify the differences in the MHR value in the subgroups of patients diagnosed with ACS or CCS. Furthermore, the differences in MHR and total serum 25(OH)D concentration between patients with diagnosis of the ACS subtypes (unstable angina (UA), non-ST elevation myocardial infarction (NSTEMI), ST-elevation myocardial infarction (STEMI)) and total serum 25(OH)D concentration were analyzed.

## 2. Materials and Methods

### 2.1. Population Characteristics

This research is based on 404 cases of male patients who received coronary angiography due to chest pain between 2013 and 2017 at Bielanski Hospital, Warsaw, Poland and consented in written form to have their data incorporated into the study. Patients with elevated inflammatory markers (elevated erythrocyte sedimentation rate and/or white blood cell count > 10,000 cells/μL, and/or CRP > 5 mg/L), viral or bacterial infection, autoimmune disease, thyroid dysfunction, active neoplasia or paraneoplastic syndromes, chronic kidney disease in stages G3–G5, as well as patients immobilized or supplementing any form of vitamin D were excluded. All patients were treated with equivalent doses of statins (40 mg of atorvastatin or 20 mg of rosuvastatin).

### 2.2. Clinical and Laboratory Data

Weight and standing height were obtained using a standard electronic scale with a telescopic measuring rod (Bielskie Wagi, Żywiec, Poland) during the admission for the computation of the body mass index (BMI, kg/m^2^). Smoking status was divided into 3 categories: smokers (yes category in the tables), ex-smokers and never-smokers (no category in the tables). To qualify as a smoker, a patient had to declare to smoke either daily or less than daily (occasionally) up to their current age and having smoked more than 100 cigarettes in a lifetime. Patients were categorized as ex-smokers if they had smoked more than 100 cigarettes and stopped smoking for at least 1 year. Never-smokers declared that they either never smoked or smoked less than 100 cigarettes in their lifetime [44]. Pre-diabetes was used to describe either impaired fasting glucose (fasting plasma glucose between 100 and 125 mg/dL) or impaired glucose tolerance (oral glucose tolerance test after 2 h between 140 and 200 mg/dL). Diabetes was diagnosed through two consecutive fasting plasma glucose measurements exceeding 126 mg/dL or a single random plasma glucose measurement exceeding 200 mg/dL with the coexistence of symptoms characteristic for hyperglycemia (polyuria, polydipsia, polyphagia) or oral glucose tolerance test after 2 h exceeding 200 mg/dL [45]. Hyperlipidemia was determined if the total cholesterol exceeded 200 mg/dL and/or triglycerides exceeded 150 mg/dL; to diagnose dyslipidemia, the concentration of LDL had to exceed 70 mg/dL and the HDL concentration did not exceed 50 mg/dL [46]. Hypertension was determined if systolic and/or diastolic blood pressure readings surpassed 140 mmHg and 90 mmHg, respectively, in at least two office measurements or when the average systolic and/or diastolic blood pressure in multiple outside office measurements exceeded 135 mmHg and 85 mmHg, respectively [47].

Laboratory measurements were carried out on fasting blood samples from the antecubital vein using standard clinical–chemical assays. Total blood count (measured to 10 April 2014 with SYSMEX XT2000i analyzer; since 11 April 2014 measured with SYSMEX XN1000 analyzer, SYSMEX, Kobe, Japan), lipid profile (measured with Cobas Integra 400 Plus, Roche Diagnostics, Rotkreuz, Switzerland), and serum 25(OH)D concentration were carried out for the calculation of MHR (monocyte count (10^3^ cells/μL) divided by HDL-C (mg/dL), and correlations between those factors were conducted in the same sample. LDL cholesterol was calculated with the Friedewald formula for patients with triglycerides under 400 mg/dL, and for the remaining patients, it was measured with a direct assay provided by the hospital laboratory. A chemiluminescent immunoassay method, DiaSorin LIAISON^®^ 25 OH Vitamin D TOTAL Assay (Stillwater, MN, USA), was used to assess serum 25(OH)D concentration (1 ng/mL = 2.5 nmol/L [48]). It is in a good agreement with Elecsys Vitamin D Total Assay [49], which was previously approved for clinical use for the Endocrine Society reference values for 25(OH)D deficiency, which are as follows: concentrations <10 ng/mL—severe deficiency, 10–20 ng/mL—moderate deficiency, 20–30 ng/mL—mild deficiency, ≥30 ng/mL—optimal values [50].

Coronary angiography was performed by radial or femoral access. The Coronary Artery Surgery Study Score (CASSS) was used to evaluate possible stenoses by three independent cardiologists, and fractional flow reserve was used to resolve uncertain cases of moderate versus severe stenosis. It uses a sum of points (0–3) to reflect the atherosclerosis of vessels. A stenosis of more than 70% in the right coronary artery, the circumflex branch, or the anterior descending branch was classified as one point, and a stenosis of more than 50% in the left main coronary artery was classified as two points.

To diagnose ACS, increased myocardial necrosis markers (especially troponin) had to be concomitant with at least one of the following: symptoms of myocardial ischemia, recent signs of ischemia or pathological Q waves on the ECG, a new loss of viable myocardium in imaging studies, a new segmental disturbance in the heart wall movement, or coronary artery thrombus on angiography [51].

### 2.3. Statistical Analysis

The normality of data distribution was tested with Shapiro–Wilk tests. Prevalence differences between groups were determined using Pearson’s chi-squared test or Fisher’s exact test. A Mann–Whitney U test was used for group comparison of results. A Kruskal–Wallis one-way analysis-of-variance-by-ranks test (or H test) followed by Dunn’s post hoc test for multiple comparisons was used to determine the dependence between more than two groups. For patients with different diagnoses, Quade Nonparametric Analysis of Covariance was performed using the Python extension for SPSS Statistics ver. 28. As potential covariates, age, BMI, hyperlipidemia, hypertension, smoking status, type 2 diabetes mellitus status, and examination date were used. Spearman’s correlation coefficient (R) was used to analyze the correlations between variables. Log transformation (ln) was performed for variables not normally distributed, and log transformed variables (25(OH)D, MHR, age and BMI) were used only in multiple regression analysis. The multiple regression analysis was employed to explore potential factors influencing the 25(OH)D concentration. Statistical significance was acknowledged when a two-sided *p*-value did not exceed 0.05. Statistica 13 (StatSoft Inc., Tulsa, OK, USA) was used to analyze the data, and GraphPad Prism 8.0 (GraphPad Software, San Diego, CA, USA) was utilized to draw figures.

## 3. Results

### 3.1. Study Population

A comprehensive description of the patients’ characteristics is presented in Table 1.

### 3.2. Difference in Selected Parameters between Patients with CCS and Patients with ACS

Significant differences were observed between patients with ACS and CCS in age, HDL, LDL, serum 25(OH)D and MHR. There was a significant disproportion of patients in smoking status and CASSS between two groups (Table 2).

### 3.3. Differences in Selected Parameters between Patients with CCS and Patients with Different Diagnoses

Differences in selected parameters between patients with different diagnoses are presented in Table 3. Patients with STEMI were significantly younger than patients with CCS and UA (*p* < 0.001 and *p* = 0.027, respectively), presented significantly higher values of TC than patients with UA (*p* = 0.002), and also had higher values of LDL than patients with CCS (*p* < 0.001) and UA (*p* = 0.001). There was a significant disproportion of patients in CASSS, hyperlipidemia, and smoking status between analyzed groups. The highest values of HDL and serum 25(OH)D were observed in patients with CCS, whereas the highest value of MHR was observed in patients with STEMI. There were no significant differences in BMI between patients with different diagnoses.

### 3.4. Analysis of Covariance for Analysis of Serum 25(OH)D and MHR

**Figure 1:**
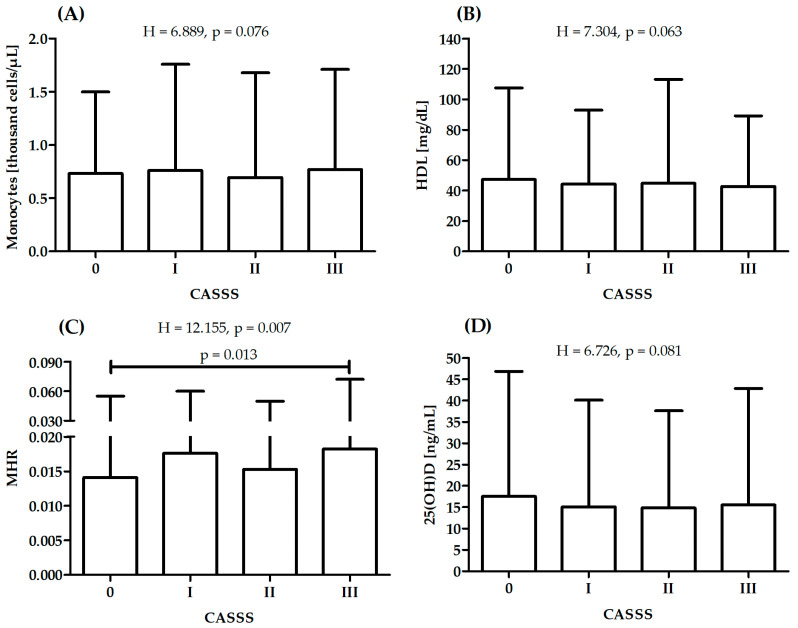
Differences in monocytes (**A**), high-density lipoprotein—HDL (**B**), monocyte-to-high-density lipoprotein ratio—MHR (**C**) and serum 25(OH)D (**D**) between patients with different CASS score: sum of points (0–III) to reflect atherosclerosis of vessels; H—Kruskal–Wallis one-way analysis-of-variance-by-ranks test.

There was a significant difference in MHR between patients with CASSS 0 and 3, while there were no significant differences between patients with different CASSS values in serum 25(OH)D, monocytes or HDL (Figure 1). After adjustment for age and hyperlipidemia status (independently), there were no significant differences in serum 25(OH)D between patients with different diagnoses (F = 1.645, *p* = 0.178 and F = 2.317, *p* = 0.075). There were no covariates that had a significant influence on results for monocytes, HDL and MHR.

### 3.5. Determinants of Serum 25(OH)D Concentration

As a result of variations in UVB sunlight availability in Warsaw, Poland (located at 52°13′ N, 21°02′ E), seasonal concentration fluctuations were noted with lower concentrations occurring from November to April compared to higher concentrations from May to October [52]. There were no significant associations between data examination and diagnosis (Chi^2^ = 0.476, *p* = 0.490). The factors influencing the natural logarithm of 25(OH)D concentration are detailed in Table 4. The proposed model proved to be statistically significant, accounting for 6% of the variance in 25(OH)D (F = 7.32, *p* < 0.001).

Table 5 presents determinants of the MHR. The proposed model was significant and explained 3% of MHR variance (F = 3.75, *p* = 0.011). BMI turned out to be a significant determinant of MHR.

### 3.6. Association between Serum 25(OH)D Concentration and Monocytes, HDL and MHR

**Figure 2:**
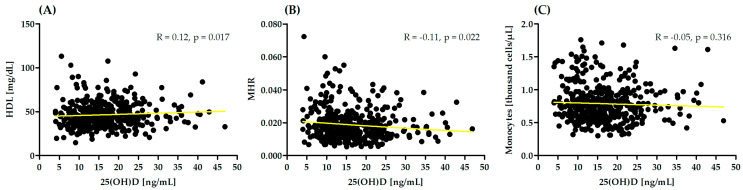
Correlation between monocytes, high-density lipoprotein (HDL) (**A**), monocyte-to-high-density lipoprotein ratio (MHR) (**B**) and serum 25(OH)D concentrations (**C**). R—Spearman correlation coefficient.

In the presented study, correlation analysis between monocytes, HDL, MHR and serum 25(OH)D concentrations was performed. Significant correlation was observed between 25(OH)D and HDL and MHR (Figure 2).

## 4. Discussion

This research describes the differences in MHR as a systemic marker of inflammation between cardiac male patients with different diagnoses. This analysis of MHR in more than 400 male patients with chest pain resulted in finding higher MHR in patients with ACS diagnosis compared to those with CCS. Investigating ACS subtypes revealed the highest MHR in patients with STEMI and the lowest MHR in patients with stable CCS. Furthermore, patients with ACS had lower serum 25(OH)D concentration compared to those with CCS, which is also inversely correlated with MHR. The mentioned differences were significant; however, they were also nominally small. The whole group presented low serum 25(OH)D concentration and high MHR values. Importantly, differences in serum 25(OH)D concentration between patients with different diagnoses could be masked by differences in age and/or hyperlipidemia status. It is a continuation of a project on a relationship between markers derived from simple laboratory tests with CCS and its complications. Recently, we observed higher markers values based on total blood count in patients diagnosed with ACS [43].

Currently, there are a limited number of studies on MHR in CVD in male patients. An analysis of nearly 900 cases of patients diagnosed with CCS revealed a significantly higher MHR in men compared to women. This marker was also found to positively correlate with a high SYNTAX score in men with CCS but not in men with ACS [9]. On the other hand, in the mixed cohort of patients, MHR was correlated with the severity of coronary atherosclerosis in CCS cases [53] as well as ACS [20]. Our analysis confirms, at least to some extent, the aforementioned correlations. However, the character of the presented cohort should be underlined, as more than 80% of the patients had at least one significant stenosis of the major coronary artery. In addition, our research used a less complicated method (CASSS compared to SYNTAX) to describe the severity of coronary atherosclerosis compared to previously mentioned articles. Nevertheless, these results suggest that the higher MHR in men diagnosed with STEMI compared to other subtypes of ACS could be related to subclinical inflammation that promotes coronary artery occlusion.

Our results show a higher MHR in men diagnosed with ACS. However, there was a very small nominal difference in the mean MHR values between numerous groups of male patients with ACS and CCS. A small *p*-value could result from a small effect and a large enough sample size [54]. The correlation between MHR in the diagnosis of ACS in patients was previously described, but it did not account for possible differences in the subgroups of men and women separately. A recent analysis of more than 100 patients revealed higher values of this marker in individuals with hypertension and unstable angina compared to those with CCS and unremarkable findings on coronary angiography [15]. A meta-analysis based on nearly 6500 patients in eight epidemiological research articles correlated MHR with a higher risk of major adverse cardiovascular events (MACE) and all-cause mortality, which suggests the predictive role of this marker [55]. Kundi et al. observed that MHR predicts MACE better than monocyte count alone [56], which corresponds with our results.

Our results showed a lower 25(OH)D concentration in men diagnosed with ACS compared to those with CCS and are consistent with the conclusions of other articles [57]. A recent analysis of patients with STEMI described low 25(OH)D levels as an independent predictor of high thrombus burden [58] and compromised coronary reperfusion [59]. Epidemiological data suggest that low 25(OH)D levels correlate with an increased incidence and morbidity due to CVD [36,60,61], but they are contrary to meta-analyses that describe the lack of benefits of 1,25(OH)D supplementation in reducing CVD-related mortality, including ACS [62]. However, in hypertensive patients, three months of 2000 IU 1,25(OH)D supplementation benefited endothelial function and a decreased expression of oxLDL and ICAM1 expression [63]. Furthermore, in critically ill patients, an early enteric supply of high-dose 1,25(OH)D was reported to be beneficial [64].

To our knowledge, these results are the first to show a negative correlation between serum 25(OH)D concentration and MHR in men with a diagnosis of CCS confirmed by coronary angiography. It should be noted that men with STEMI were found to have the lowest 25(OH)D concentration. The correlation between serum 25(OH)D and MHR was previously described by Mousa et al., but their cohort comprised 860 young, healthy adults [65]. What is more, vitamin D deficiency was found in young men with normal body weight with atherogenic lipid profile [66]. Despite the results of multiple observational studies suggesting the correlation between vitamin D and HDL concentration, their causal relationship is not described yet [67,68]. Some recent investigations point toward the possible role of proteomics in the mechanisms connecting vitamin D, lipid metabolism and inflammation [69]. Apolipoproteins, less often measured structural components of lipoprotein particles, were suggested to be a missing link between vitamin D and its influence on lipid metabolism [70]. Calcitriol was described to stimulate apolipoprotein A1 production and reverse cholesterol transport, which results in HDL concentration increase [70,71].

In addition to the mentioned correlation between lipid profile, 1,25(OH)D modulates immune system function through a variety of cells, including monocytes and macrophages [72]. The phenotype of macrophages plays a key role in the stability of atherosclerotic plaque. Recent studies in mice showed that under UVB, macrophages are promoted toward the anti-inflammatory phenotype of M2, which is suggested to be a new way to decrease inflammation and stabilize plaques [73]. In advanced atherosclerosis, macrophages were reported to have autophagy dysfunctions that are suggested to be regulated by calcitriol. Furthermore, 1,25(OH)D was found to significantly decrease oxidized LDL-impaired autophagy, increase autophagy-mediated lipid breakdown in human monocyte-derived macrophages, and prevent them from becoming foam cells [74].

The mechanism of calcitriol’s influence on HDL cholesterol fractions and monocyte functions presented above may explain the correlation between low 25(OH)D concentration and higher MHR observed in the presented study. However, taking into account similar results in other studies, one should consider the possibility of bidirectional linkage between these variables, where MHR is a predictor for vitamin D concentration [75,76].

This research has its limitations. The cross-sectional and observational character of this study ruled out the investigation of the causal relationship and innovative analysis of the mechanism underlying the association between measured variables. The subgroups of patients with different diagnoses were not matched. The number of cases analyzed was limited and only included Polish male patients. The biochemical analysis did not include inflammatory cytokines, ferritin, glycated hemoglobin level, HOMA IR index and calcitriol, as it was based on cholecalciferol concentrations alone. In addition, a body composition analysis was not included. The extent of atherosclerosis was described using a simple CASSS scale; the impact of calcifications on vessels, the type of statin ingested, and the length of statin therapy were not taken into account.

The currently available data as well as the results of our study suggest that inflammation plays a key role in the initiation, progression and especially destabilization of atherosclerotic plaque leading to myocardial infarction. This implies that new inflammatory markers could allow the identification of patients with a higher risk of MACE. The correlation between MHR values and the diagnosis of ACS in men could be used as an additional tool to predict ACS in male patients with chest pain, but this needs to be confirmed in further well-designed studies. In our opinion, the small but significant differences in the value of the subclinical inflammatory marker shown in our study indicate the need to focus the treatment of CCS on immunosuppressive effects and to search for drugs with such a systemic mechanism of action. The lower concentration of 25(OH)D in men with ACS and its correlation with MHR suggest a probable relationship between this vitamin, which requires further longitudinal studies, as vitamin D has a well-documented effect on inflammation, and acute complications of atherosclerosis. However, a bidirectional relationship between MHR and 25(OH)D levels should also be considered, where an elevated monocyte/HDL ratio may be a predictor of vitamin D deficiency. Considering the predicted high usefulness of MHR as an indicator of vitamin D supply in outpatient care in less affluent countries, we believe that it requires urgent verification and should be urgently clarified. In addition, it may be possible to conduct further large studies based on the MHR value and vitamin D level to identify male patients who require supplementation with higher doses of vitamin D or the implementation of recommendations regarding the duration of exposure to solar radiation or modification of eating habits.

We suggest that MHR should be a subject of longitudinal research to assess its possible value as a more comprehensive additional marker that could simultaneously express inflammation and lipid metabolism as an additional factor that prognoses ACS in men with chest pain. In male cardiac patients, the MHR could also be an indicator of the vitamin D supply status. Nevertheless, there is a need for more comprehensive and well-designed randomized studies to fully assess the role of MHR and 25(OH)D in atherosclerosis and its complications.

## 5. Conclusions

Men referred for coronary angiography due to chest pain and diagnosed with ACS were found to have higher MHR and lower 25(OH)D concentration than male patients with CCS. However, very small nominal differences in mean MHR values were observed between subgroups of patients with ACS and CCS. Male patients with STEMI had the highest MHR. The lowest 25(OH)D levels were observed in patients with STEMI compared to those diagnosed with CCS; however, the effect was associated with significant age differences between groups. MHR and 25(OH)D were negatively correlated in the whole group. These results suggest the need for further well-designed research to determine the role of vitamin D and MHR in the acute complications of atherosclerosis and stratification of ACS risk in men with chest pain. Increasing the number of patients included in the study would allow researchers to better assess the relationship between MHR and the diagnosis of ACS. It will also allow determin the importance of the MHR value as a predictor of the body’s supply of vitamin D. Furthermore, the analysis of subgroups matched in terms of age and lipid metabolism would expand our understanding of the possible correlation between the level of vitamin D and the diagnosis of ACS and CCS.

## Figures and Tables

**Table 1:** Characteristics of participants.

Variable	Values
Age (years)	63.3 (30.9–93.3)
BMI (kg/m^2^)	27.7 (16.1–47.4)
BMI classification (<25 kg/m^2^/25–29.9 kg/m^2^/>30 kg/m^2^)	93/173/109
Cause of hospitalization (CCS/ACS)	206/198
Previous MI (yes/no)	168/236
CASSS (0/1/2/3)	72/115/118/99
Total cholesterol (mg/dL)	167 (70–334)
High-density lipoprotein (mg/dL)	44.6 (14.6–113.2)
Low-density lipoprotein (mg/dL)	94.1 (22.3–257.9)
Triglycerides (mg/dL)	112 (37–457)
Hyperlipidemia (yes/no)	234/170
Hypertension (yes/no)	331/73
Smoking (active/former smoker/no)	138/53/213
Type 2 diabetes mellitus (yes/pre-diabetes/no)	139/17/248
Monocytes (thousand cells/µL)	0.74 (0.30–1.76)
MHR	0.02 (0.01–0.07)
Serum 25(OH)D (ng/mL)	15.6 (4.0–46.9)
Examination date (May–October/November–April)	106/298

Data for the variables presented as number or median (range). BMI—body mass index; CCS—chronic coronary syndrome; ACS—acute coronary syndrome; MI—myocardial infarction; CASSS—Coronary Artery Surgery Study Score; MHR—monocyte-to-high-density lipoprotein ratio.

**Table 2:** Differences in selected parameters between patients with ACS and CCS.

Variable	ACS	CCS	*p*-Value
*N*	198	206	-
Age (years)	61.9 (33.6–88.4)	64.3 (30.9–93.3)	<0.001
BMI (kg/m^2^)	27.3 (16.9–44.6)	27.8 (16.1–47.4)	0.30
Previous MI (no/yes)	106/92	130/76	0.05
CASSS (0/1/2/3)	10/72/61/55	62/43/57/44	<0.001
Total cholesterol (mg/dL)	170 (70–334)	163.8 (96.0–327.3)	0.25
High-density lipoprotein (mg/dL)	42.5 (19.5–92.9)	46.0 (14.6–113.2)	<0.001
Low-density lipoprotein (mg/dL)	100 (24–244)	86.2 (22.3–257.9)	0.014
Triglycerides (mg/dL)	112 (43–457)	113.0 (37.1–438.3)	0.52
Hyperlipidemia (no/yes)	79/119	91/115	0.38
Hypertension (no/yes)	29/169	44/162	0.08
Smoking (no/ex-smokers/yes)	94/16/88	119/37/50	<0.001
Type 2 diabetes mellitus (yes/pre-diabetes/no)	130/7/60	116/10/79	0.15
Monocytes (thousand cells/µL)	0.76 (0.31–1.65)	0.73 (0.30–1.76)	0.27
MHR	0.018 (0.01–0.07)	0.015 (0.01–0.06)	<0.001
Serum 25(OH)D (ng/mL)	14.2 (4.0–42.9)	16.8 (4.1–46.9)	0.010
Examination date (May–October/November–April)	55/143	51/155	0.49

Data presented as number or median (range). CCS—chronic coronary syndrome; ACS—acute coronary syndrome; BMI—body mass index; MI—myocardial infarction; CASSS—Coronary Artery Surgery Study Score; MHR—monocyte-to-high-density lipoprotein ratio; prevalence differences between groups were determined using Pearson’s chi-squared test or Fisher’s exact test; Mann–Whitney U test was used for group comparison of results.

**Table 3:** Differences in selected parameters between patients with different diagnoses.

Variable	CCS	STEMI	NSTEMI	UA	*p*-Value
*N*	206	107	59	32	-
Age (years)	64.3 (30.9–93.3)	59.8 (36.3–88.4)	61.5 (38.0–86.4)	66.5 (33.6–80.8)	<0.001
BMI (kg/m^2^)	27.8 (16.1–47.4)	27.0 (16.9–44.6)	27.9 (20.8–36.1)	26.4 (22.2–34.6)	0.24
Previous MI (no/yes)	130/76	60/47	26/33	20.12	0.06
CASSS (0/1/2/3)	62/43/57/44	3/50/31/23	5/17/19/18	2/5/11/14	<0.001
Total cholesterol (mg/dL)	164 (96–327)	180 (98–320)	165 (70–334)	144 (81– 303)	0.002
High-density lipoprotein (mg/dL)	46.0 (14.6–113)	44.3 (20.5–92.9)	39.3 (22.8–73.2)	42.8 (19.5–65.8)	<0.001
Low-density lipoprotein (mg/dL)	86.2 (22.3–258)	107 (38.2–214)	96.4 (23.5–244)	75.5 (32.9–228)	<0.001
Triglycerides (mg/dL)	113 (37–438)	108 (55–368)	115 (44–457)	109 (43–252)	0.49
Hyperlipidemia (no/yes)	91/115	35/72	23/36	21/11	0.008
Hypertension (no/yes)	44/162	20/87	8/51	1/31	0.07
Smoking (no/ex-smokers/yes)	119/37/50	45/7/55	27/3/29	22/6/4	<0.001
Type 2 diabetes mellitus (yes/pre-diabetes/no)	116/10/79	75/3/28	37/4/18	18/0/14	0.15
Monocytes (thousand cells/µL)	0.73 (0.30–1.76)	0.80 (0.31–1.63)	0.68 (0.40–1.65)	0.76 (0.31–1.54)	0.06
MHR	0.015 (0.01–0.06)	0.018 (0.01–0.06)	0.017 (0.01–0.05)	0.018 (0.01–0.07)	0.009
Serum 25(OH)D (ng/mL)	16.8 (4.1–46.9)	14.1 (4.0–42.9)	14.0 (5.0–37.6)	17.6 (4.3–36.7)	0.035
Examination date (May–October/November–April)	51/155	28/79	17/42	10/22	0.84

Data presented as number or median (range). CCS—chronic coronary syndrome; BMI—body mass index; MI—myocardial infarction; CASSS—Coronary Artery Surgery Study Score; MHR—monocyte-to-high-density lipoprotein ratio; prevalence differences between groups were determined using Pearson’s chi-squared test or Fisher’s exact test; Kruskal–Wallis one-way ANOVA followed by Dunn’s post hoc test for multiple comparisons was used to determine the dependence between groups.

**Table 4:** Factors influencing ln25(OH)D concentration.

Determinants	β (SE)	*p*-Value
Age	0.13 (0.05)	0.009
BMI	0.07 (0.05)	0.15
Examination date (May–October/November–April)	−0.19 (0.05)	<0.001

SE—standard error; BMI—body mass index; examination date is categorical variable: May–October—0, November–April—1.

**Table 5:** Factors influencing MHR.

Determinants	β (SE)	*p*-Value
Age	−0.05 (0.05)	0.31
BMI	0.14 (0.05)	0.006
Examination date (May–October/November–April)	0.08 (0.05)	0.144

SE—standard error; BMI—body mass index; examination date is categorical variable: May–October—0, November–April—1.

## Data Availability

Data can be provided by the corresponding author upon reasonable request.
